# Emergence of two distinct phase transitions in monolayer CoSe_2_ on graphene

**DOI:** 10.1186/s40580-024-00427-4

**Published:** 2024-05-24

**Authors:** Tae Gyu Rhee, Nguyen Huu Lam, Yeong Gwang Kim, Minseon Gu, Jinwoong Hwang, Aaron Bostwick, Sung-Kwan Mo, Seung-Hyun Chun, Jungdae Kim, Young Jun Chang, Byoung Ki Choi

**Affiliations:** 1 Department of Physics, University of Seoul, Seoul, 02504 Korea; 2 Department of Smart Cities, University of Seoul, Seoul, 02504 Korea; 3 Department of Physics, University of Ulsan, Ulsan, 44610 Korea; 4 Department of Physics, Institute of Quantum Convergence Technology, Kangwon National University, Chuncheon, 24341 Korea; 5 Advanced Light Source, Lawrence Berkeley National Laboratory, Berkeley, CA 94720 USA; 6 Department of Physics, Sejong University, Seoul, 05006 Korea; 7 Department of Intelligent Semiconductor Engineering, University of Seoul, Seoul, 02504 Korea

**Keywords:** Transition metal chalcogenides, Charge-density wave, Electron-boson coupling, Molecular beam epitaxy, Angle-resolved photoemission spectroscopy, Scanning tunneling microscopy

## Abstract

Dimensional modifications play a crucial role in various applications, especially in the context of device miniaturization, giving rise to novel quantum phenomena. The many-body dynamics induced by dimensional modifications, including electron-electron, electron-phonon, electron-magnon and electron-plasmon coupling, are known to significantly affect the atomic and electronic properties of the materials. By reducing the dimensionality of orthorhombic CoSe_2_ and forming heterostructure with bilayer graphene using molecular beam epitaxy, we unveil the emergence of two types of phase transitions through angle-resolved photoemission spectroscopy and scanning tunneling microscopy measurements. We disclose that the 2 × 1 superstructure is associated with charge density wave induced by Fermi surface nesting, characterized by a transition temperature of 340 K. Additionally, another phase transition at temperature of 160 K based on temperature dependent gap evolution are observed with renormalized electronic structure induced by electron-boson coupling. These discoveries of the electronic and atomic modifications, influenced by electron-electron and electron-boson interactions, underscore that many-body physics play significant roles in understanding low-dimensional properties of non-van der Waals Co-chalcogenides and related heterostructures.

**Graphical Abstract:**

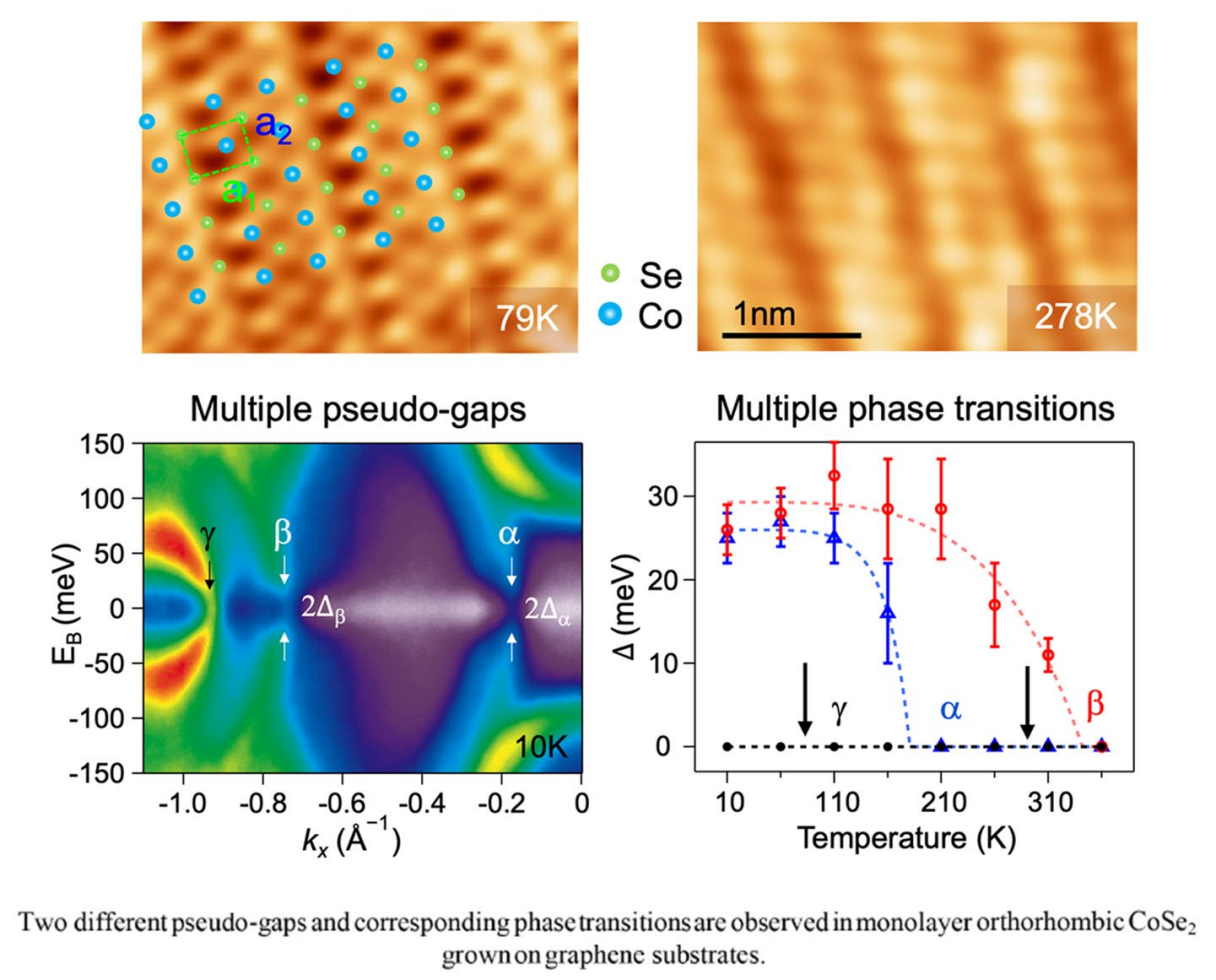

**Supplementary Information:**

The online version contains supplementary material available at 10.1186/s40580-024-00427-4.

## Introduction

Atomically thin films have become pivotal in numerous applications and fundamental research studies [1–4]. The incessant drive for device miniaturization necessitates an exploration of the electronic properties of atomically thin films, distinctly different from their bulk counterparts. In the realm of dimensionality reduction, the material’s electronic and atomic structure can manifest new ordering phenomena that remain unexplored in the bulk form. The charge density wave (CDW) stands out as one of the most studied correlated phenomena, characterized by static structural and electronic ordered states. Diminishing dimensions lead to the emergence of novel CDW phenomena or projected CDW inherited from bulk counterpart, which are concomitant with enhancements in Fermi surface (FS) nesting [5–7], electron-phonon coupling (EPC) [7–10], excitonic interaction [11–13], and electronic correlation [13–15], along with a concurrent suppression of electronic screening. Numerous studies propose that CDW orders bear relevance to superconductivity [16–20] or magnetism [21–24], representing a captivating topic in solid-state physics. Materials exhibiting CDW or high-temperature superconductivity share common features absent in conventional metals. These features encompass a temperature-dependent Hall coefficient [25], pseudo-gap [26, 27], and EPC [28, 29]. In addition to CDW, the interfacial effects with substrates emerge as crucial characteristics resulting from dimensional reduction [30, 31]. These effects influence the system through charge transfer, lattice stress or many-body interactions, constituting an important aspect for both research and device applications.

Despite the intensive studies of electronic and structural properties associated with dimensional modifications in quasi-two-dimensional (2D) transition metal chalcogenides (TMC) with van der Waals (vdW) stacking, such as V-, Ta-, Ti-, Ir- and Nb-chalcogenides [6, 10–12, 32–37], non-vdW TMCs have been scarcely investigated. As proposed in the literature on 2D non-vdW materials, such as iron ore hematite (Fe_2_O_3_), MnSe_2_, and FeS_2_, the synthesis of 2D non-vdW materials is likely to yield significantly different electronic structures compared to their bulk counterparts [38–41]. This discrepancy is primarily attributed to its heightened sensitivity to sample thickness, which is higher compared to that of vdW materials. Cobalt-based chalcogenides, belonging to the TMC family, exhibit diverse stoichiometric forms based on the growth temperatures [42]. Cobalt selenides have several polymorphs, such as orthorhombic CoSe_2_ (O-CoSe_2_) [43], cubic CoSe_2_ [44], hexagonal Co_2_Se_3_ [45], 1T CoSe_2_ [46], hexagonal CoSe [47], and tetragonal CoSe [46]. Among them, O-CoSe_2_ has intriguing properties, positioning it as a promising candidate for potential applications in catalysts, flexible energy storages, antibacterial applications [43, 48–50]. Despite its potential, the electronic structure of O-CoSe_2_ has yet to be studied. Therefore, O-CoSe_2_ may serve as a suitable material for investigating the effects of dimensional modifications. While TMC materials with 1T or 2 H phases are stacked with van der Waals bonding, the layers of O-CoSe_2_ are tightly stacked together by non-vdW interaction. This implies that more dramatic electronic effects are expected for the non-vdW materials when transitioning from 3D to 2D [38]. 

Here, we report the successful growth of monolayer (ML) O-CoSe_2_ on the bilayer graphene (BLG) on 4H-SiC substrates using molecular beam epitaxy (MBE). We conducted a comprehensive investigation of the electronic and atomic structure of ML O-CoSe_2_ employing scanning tunneling microscopy (STM) and angle-resolved photoemission spectroscopy (ARPES). Our observations reveal that ML O-CoSe_2_ simultaneously exhibits two emergent phase transitions, which have distinct transition temperatures determined by fitting the temperature-dependent pseudo-gap size with Dynes formula [51, 52]. The phase transition with 2 × 1 superstructure involves FS nesting accompanied by a pseudo-gap. However, another phase transition, which cannot be explained by FS nesting due to its lack of a nesting region in the FS, is coupled with electron-boson coupling (EBC).

## Methods

CoSe_2_ films were grown using a custom-built MBE chamber with a base pressure of 1 × 10^− 10^ Torr. The 4H-SiC (001) single-crystal substrates, provided by the Crystal Bank at Pusan National University, was degassed at 600 ℃ for 12 h, and underwent annealing at 1400 ℃ for 2 min three times under ultrahigh vacuum (UHV) conditions to grow a BLG layer on the Si-terminated surface of 4H-SiC. The detailed analysis of the number of graphene layers is illustrated in Figure S1. Co (99.995%) and Se (99.999%) were co-evaporated using an e-beam evaporator and an effusion cell, respectively [35, 53, 54]. During film growth, the substrate temperature was maintained at 250 ℃ for 10 min duration to achieve a film thickness of 1 ML thickness. *In situ* RHEED measurements were performed with a high voltage of 18 kV. For both STM and ARPES measurements, the sample were covered with an amorphous selenium layer at room temperature to protect the pristine surface from air exposure after the film growth. Subsequently, the samples underwent annealing at 520 K in UHV to remove the selenium capping layer. STM measurements were carried out in a home-built STM under a base pressure of ∼7 × 10^− 11^ Torr at 79 K and 290 K [55]. STM topography was taken at constant-current mode with the bias voltage applied to the sample. Tungsten tips were electrochemically etched and cleaned *in situ* by electron beam heating. ARPES data were acquired using a Scienta R4000 analyzer at the base pressure 3 × 10^− 11^ Torr in APRES endstation at Beamline 10.0.1 at the Advanced Light Source, Lawrence Berkeley National Laboratory. The main data sets were obtained with a photon energy set at 63 eV, featuring energy and angular resolution of 18–25 meV and 0.1°, respectively. The intensity of the measured spectral features strongly varies depending on the polarization due to the photoemission matrix element [56, 57]. The *k*_z_ dependence data were acquired with various photon energies in the range of 40–80 eV. XPS data were obtained at Beamline 8A2 (KBSI-PAL APXPS) at the Pohang Light Source.

## Results and discussion

**Fig. 1:**
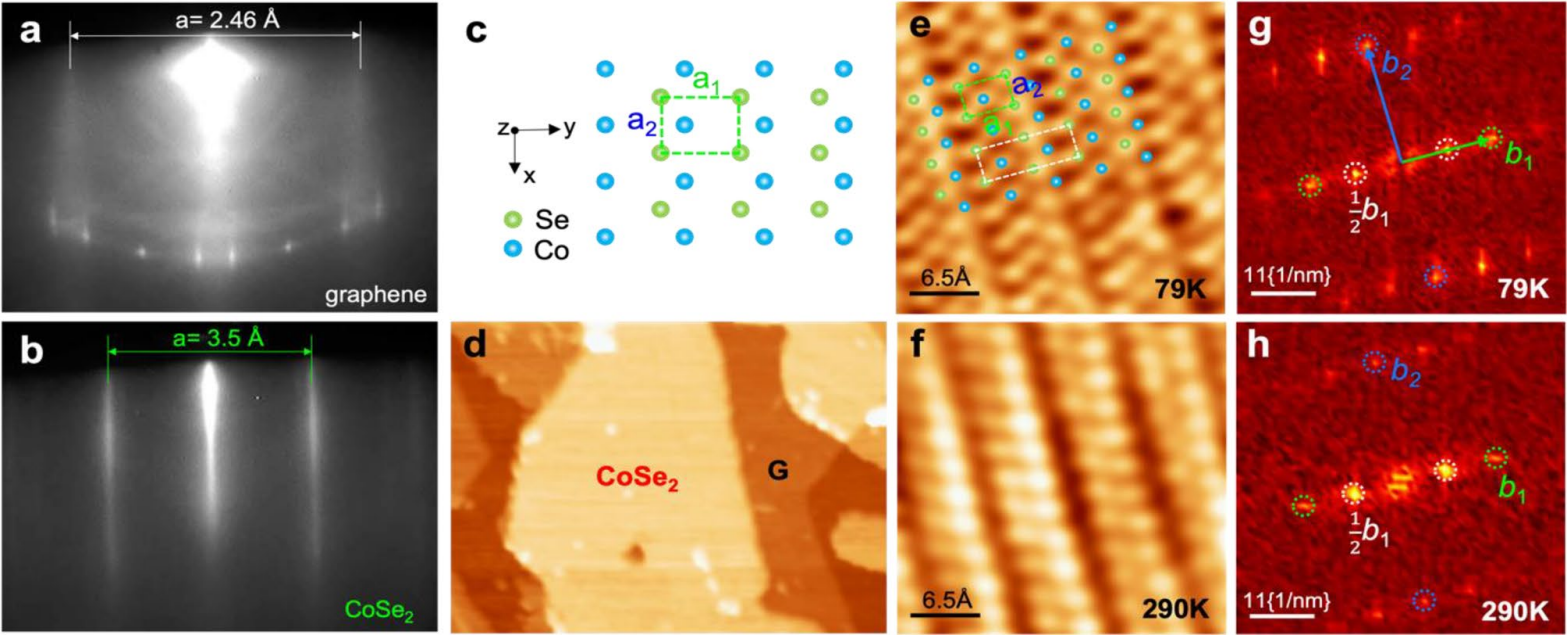
Atomic structure of ML O-CoSe_2_ film. (**a**,**b**) *In situ* RHEED images of BLG on SiC and ML O-CoSe_2_ on BLG grown by MBE. (**c**) Top view schematics of CoSe_2_ with orthorhombic structure. The sky-blue and dark-green balls represent the Co and Se atoms, respectively. (**d**) Large-scale topographic STM image of ML O-CoSe_2_ grown on BLG at 79 K. (**e**–**h**) Atomically resolved STM images (**e, f**) and corresponding FFT images (**g**,**h**) of ML O-CoSe_2_ grown on BLG at 79 K and 290 K. The *b*_1_ and *b*_2_ are orthorhombic lattice peaks. $\frac{1}{2}{b}_{1}$ represents CDW peaks marked by white circles. Scanning conditions: (d) *V*_b_ = 3.0 V, *I*_t_ = 30 pA; (e) *V*_b_ = -0.2 V, *I*_t_ = 40 pA; and (f) *V*_b_ = 2.0 V, *I*_t_ = 30 pA

**Fig. 2:**
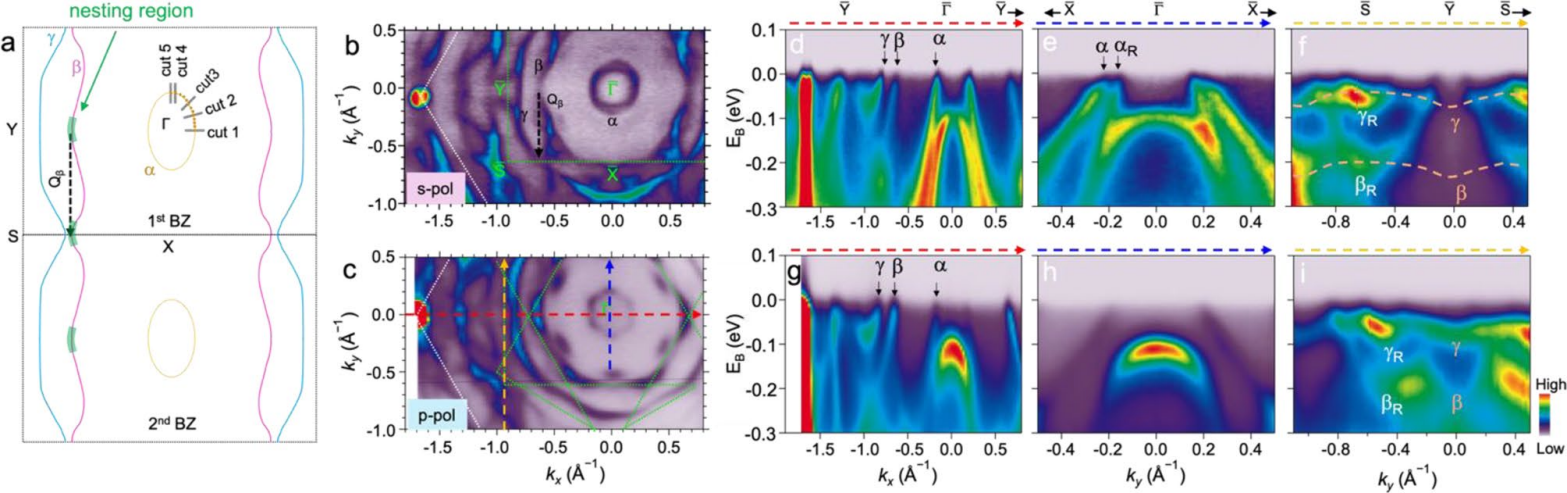
Electronic structure of ML O-CoSe_2_ using ARPES measurements with *s*- and *p*-polarizations at 10 K. **(a)** A schematic of Fermi surface (FS) of ML O-CoSe_2_. The green shadow areas represent nesting regions. **(b, c**) FS contour and (**d-i**) ARPES maps of ML O-CoSe_2_ taken along $\stackrel{-}{\text{Y}}$−$\stackrel{-}{{\Gamma }}$−$\stackrel{-}{\text{Y}}$ (**d, g**), $\stackrel{-}{\text{X}}$−$\stackrel{-}{{\Gamma }}$−$\stackrel{-}{\text{X}}$ (**e, h**) and $\stackrel{-}{\text{S}}$−$\stackrel{-}{\text{Y}}$−$\stackrel{-}{\text{S}}$ (**f, i**) directions using *s*-polarized (**d-f**) and *p*-polarized (**g-i**) photon sources. The α, β, and γ indicate the three main bands, while the α_R_, β_R_, and γ_R_ indicate 120° rotated main bands. ${Q}_{\beta }$ vector presents a nesting vector related to the β bands

Figure 1a and b present the reflection high-energy electron diffraction (RHEED) patterns of the BLG/SiC substrate (top) and the ML O-CoSe_2_ (bottom), showing sharp vertical line profiles maintained during the co-deposition of Co and Se. This observation suggests a uniform and well-ordered film formation with an in-plane lattice constant of 3.5 ± 0.05 Å, referencing the lattice constant of graphene (2.46 Å) (Figure S2). The stoichiometry of the film is verified through x-ray photoemission spectroscopy (XPS) analysis, as depicted in Figure S3. The high-quality morphology of the ML O-CoSe_2_ film is further confirmed by a large-scale STM topographic image in Fig. 1d. In Fig. 1e obtained at 79 K, atomically resolved STM images of ML O-CoSe_2_ reveal an orthorhombic unit cell, as illustrated in Fig. 1c, (green box) with stripe modulations showing 2 × 1 periodicity (white box). Corresponding fast Fourier transformation (FFT) image of Fig. 1g clearly exhibits that the orthorhombic lattice peaks of b_1_, b_2_, and 2 × 1 modulation peaks of 1/2 b_1_, which has not been observed in bulk O-CoSe_2_ [42]. The lattice constants of the ML O-CoSe_2_ are determined as *a*_1_ = 4.77 Å and *a*_2_ = 3.54 Å, agreeing with the values from RHEED analysis and the reported bulk values of *a*_1_ = 4.849 Å and *a*_2_ = 3.6 Å [42]. Interestingly, the strong 2 × 1 modulation is well preserved at room temperature (290 K) as shown in Fig. 1f and h. It is indeed suggested as an emergent CDW phase of ML O-CoSe_2_, absent in the bulk, whose origin will be further discussed in Fig. 2.

The most common features of the CDW in electronic structure, which give rise to atomic modulations, are electronic pseudo gap and band renormalization. These features may exhibit significant anisotropy in momentum space during the CDW transition, making ARPES the preferred choice for investigating electronic structure with momentum resolution. Taking this into account, we conducted polarization-dependent ARPES measurements to explore the CDW features in the electronic structure. Figure 2b and c present the ARPES FS maps of ML O-CoSe_2_ obtained at 10 K with *s*- and *p*-polarized light sources, respectively. While STM can probe the local surface area of films, ARPES explores a relatively large surface area due to the photon beam size (10 × 10 μm²), inevitably leading us to measure the electronic structure of multiple domains within the film. Consequently, our ARPES data encompass three distinct domains, resulting from superimposed orthorhombic symmetry on hexagonal symmetry. Three energetically equivalent domains are observed with an anisotropic crystal axis rotated by 120 degrees in relation to one another (Figure S4) [54, 58, 59]. The three mixed domains result in a star-like shaped Brillouin zone (BZ) from the superimposed three orthorhombic BZ, as illustrated in Fig. 2c with green dashed lines. The BZ of BLG is illustrated with white dashed lines.

We assigned three main bands as α, β, and γ, respectively, as shown in schematic of FS and ARPES map in Fig. 2a, b. The FS maps reveal small spectral weight on the α and β bands, suggesting the formation of pseudo-gaps at the Fermi level for those bands. The detailed gap profile of the β band is illustrated in supplementary Figure S5. These pseudo-gap formations exhibit significant anisotropy in momentum space, which is a typical phenomenon in CDW transitions [5, 6, 60, 61]. We find that the 2 × 1 modulation observed in STM satisfies the nesting condition of the β bands. In Fig. 2a, b, the nesting vector connecting pseudo-gaps of the β bands (black dashed arrows ${Q}_{\beta }$) indeed corresponds to the reciprocal vector ($\frac{1}{2}{b}_{1}$) of the 2 × 1 modulation; i.e.,

$${Q_\beta } = {1 \over 2}{b_1} = \left( {0.641\,{{\mathop {\rm{A}}\limits^ \circ }^{ - 1}}} \right){\hat b_1}$$

Therefore, it is suggested that the 2 × 1 superstructure is attributed to a CDW phase originated from FS nesting at the β bands. We note that the β band is newly formed compared to the bulk O-CoSe_2_ in the density-functional theory calculations [62]. On the other hand, the pseudo-gaps at the α bands lack a nesting region due to topology of FS. Thus, the pseudo-gap in the α band cannot be explained by FS nesting, and corresponding atomic modulations are not observed in STM data. Further discussion of pseudo-gap in the α bands will be provided in the later discussion.

Figure 2d and g depict ARPES intensity maps along the $\stackrel{-}{\text{Y}}$−$\stackrel{-}{{\Gamma }}$−$\stackrel{-}{\text{Y}}$ direction in a single BZ shown as the green dashed rectangular box in Fig. 2b with *s*- and *p*-polarized light sources, respectively. The α band is a hole pocket centered at the $\stackrel{-}{{\Gamma }}$ point, and the β and γ bands are electron pockets centered at the $\stackrel{-}{\text{Y}}$ point, with all three bands crossing the Fermi energy. Intensities of the α and β bands vary significantly depending on the photon polarization, indicating different orbital contributions [54]. Along the $\stackrel{-}{\text{X}}$−$\stackrel{-}{{\Gamma }}$−$\stackrel{-}{\text{X}}$ direction, we observed another α band from differently rotated domains, as shown in Fig. 2e, marked by ${\alpha }_{R}$, indicating its anisotropic velocities. As seen in Fig. 2f and i, flattened bands are observed along the BZ edge, marked by orange dashed lines, attributed to quasi-1D modulations in our system [54, 63]. These flat bands are observed exclusively along the S̅−Y̅−S̅ direction, which is parallel to the direction of ${Q}_{\beta }$ and atomic modulations. Additionally, we verified the 2D characteristics of ML O-CoSe_2_ through band mapping along *k*_*z*_, achieved by scanning the incident photon energy from 40 eV to 80 eV, displaying absence of dispersive feature (Figure S6).

**Fig. 3:**
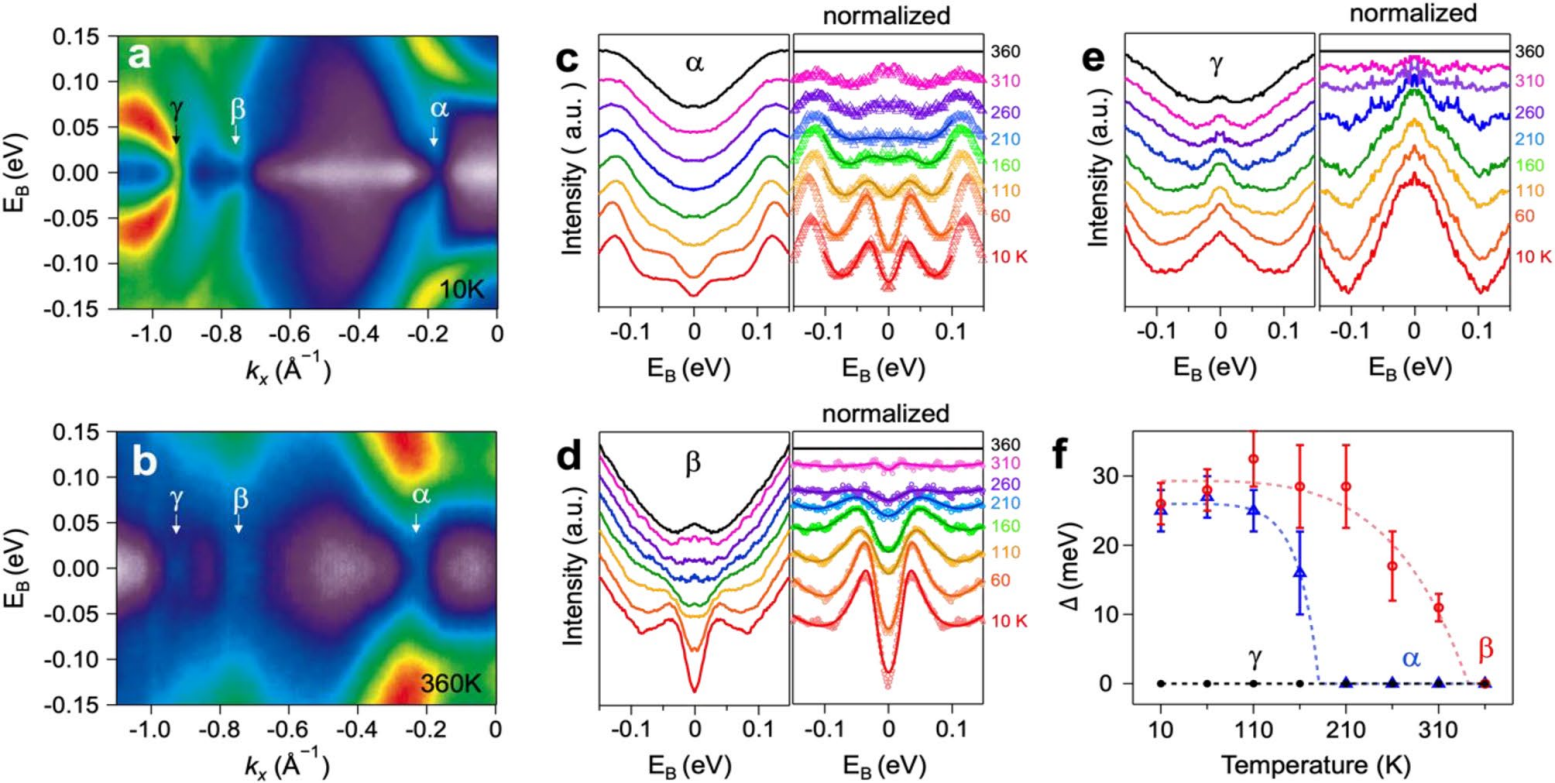
Temperature-dependent CDW gaps of ML O-CoSe_2_. (**a, b**) Symmetrized ARPES maps about the Fermi level at 10 K and 360 K, respectively. (**c-e**) Symmetrized EDCs of gap position for the α, β, and γ bands at the selected temperatures ranging from 10 K to 360 K. (**f**) The gap values (Δ) obtained from the symmetrized EDCs using the Dynes formula are fitted to a mean-field theory for the α (blue) and β (red) bands

In order to clearly delineate the pseudo-gaps in both α and β bands of ML O-CoSe_2_, ARPES maps symmetrized in energy at the Fermi level were plotted along the $\stackrel{-}{\text{Y}}$−$\stackrel{-}{{\Gamma }}$ direction for *s*-polarization at both 10 K and 360 K (Fig. 3a and b), respectively (Symmetrized ARPES maps for *p*-polarization are illustrated in Figure S7). Although the γ band remains closed at the Fermi level across all temperature ranges, both the α and β bands are open at the Fermi level at 10 K (Fig. 3a) and closed at 360 K (Fig. 3b), indicating that the temperature of 360 K exceeds the transition temperatures (FS maps for high temperature are illustrated in Figure S8). To characterize the transition temperatures, of particular interest, we monitored the temperature-dependent symmetrized energy dispersion curves (EDCs) for the three main bands during heating (Fig. 3c-e). The normalized data are scaled with respect to the data at 360 K. The dips observed in the normalized, symmetrized EDCs indicate the presence of a gap in specific momentums at the Fermi level. The gaps extracted from these EDCs using Dynes formula multiplied by Lorentzian with binomial background (Figure S9) are plotted as function of temperature in Fig. 3f:

$$I\left(\omega \right)=\left|\text{Re}\left(\frac{\omega +i{\Gamma }}{\sqrt{{\left(\omega +i{\Gamma }\right)}^{2}-{{\Delta }}^{2}}}\right)\right|\cdot \left(\frac{a}{{\omega }^{2}+{a}^{2}}+b{\omega }^{2}+c\right)+y$$

where ${\Gamma }$ is the pair-breaking scattering rate and ${\Delta }$ is the pseudo-gap [51, 52]. The gaps extracted in the ground state (10 K) using the Dynes formula are determined to be 25 meV and 28 meV for the α and β bands, respectively, and they decrease as the temperature increases. The gaps extracted for selected temperatures are fitted to the mean-field theory for second-order phase transitions [5, 11], as indicated by dashed lines:

$$\varDelta \left(T\right)\propto {tanh}^{2}\left(A\sqrt{\frac{{T}_{C}}{T}-1}\right) {\Theta }\left({T}_{C}-T\right).$$.

Here, *A* = 1.28 and 1.45 represent proportional constants for the α and β bands, respectively. ${\Theta }$ is the unit step function. The gaps in the both α and β bands are well-matched with the mean-field theory, as illustrated in Fig. 3f. As the ML O-CoSe_2_ film is heated from 10 K, the gap in the α band closes at 160 K (${T}_{C}^{\alpha }$) and the gap in the β band closes at 340 K (${T}_{C}^{\beta }$). The persistence of the gap in the β band even above 300 K suggests a robust CDW, consistent with the observed 2 × 1 superstructure measured at 290 K in the STM measurements. Although the gap in the α band is fitted with the mean-field theory, the corresponding atomic modulations are not observed in the STM results at low temperature, implying that the origin of pseudo-gap in α band differs from that of β band.

**Fig. 4:**
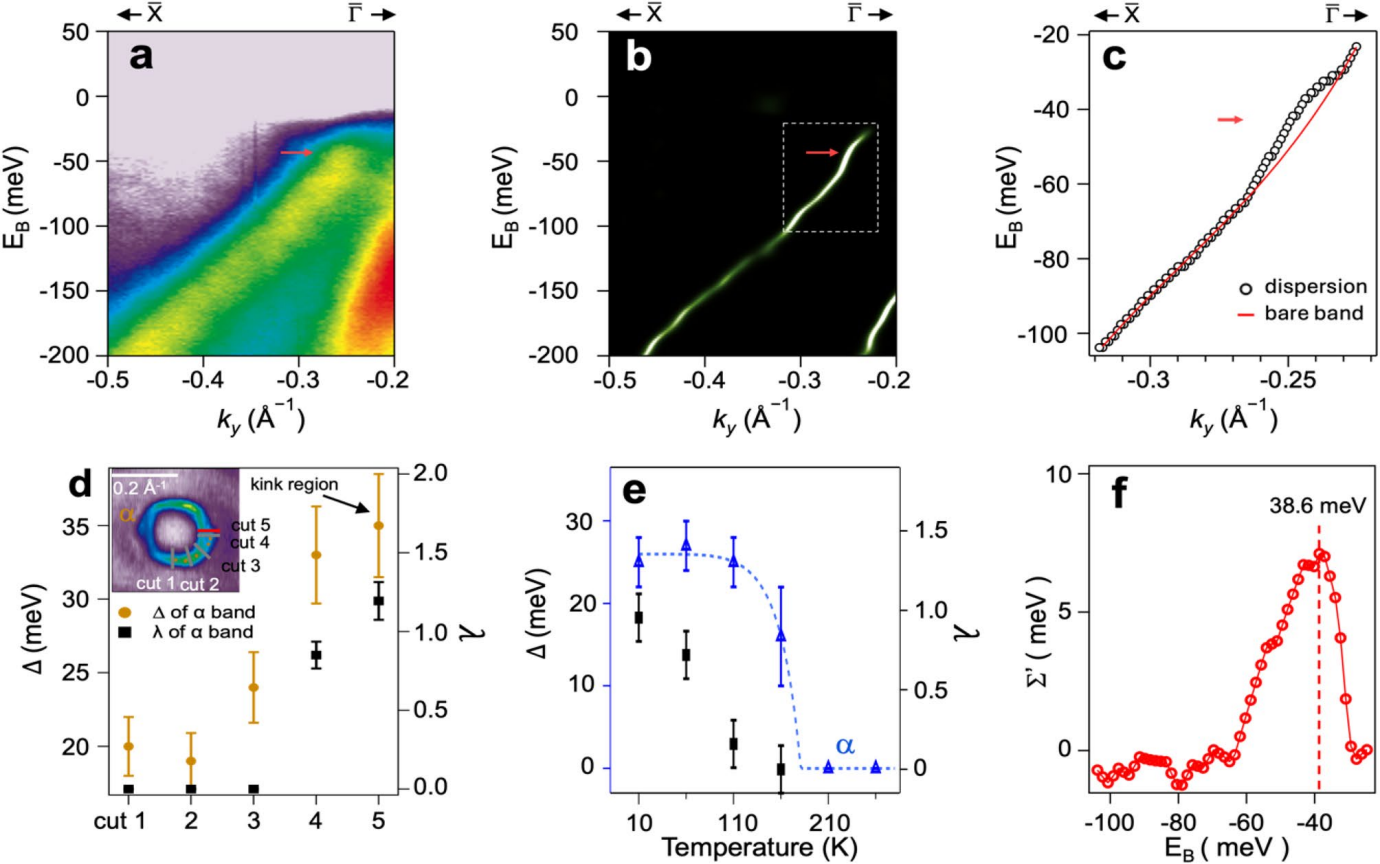
EBC in ML O-CoSe_2_. (**a**) ARPES map along the $\stackrel{-}{\text{X}}$−$\stackrel{-}{{\Gamma }}$ direction at 10 K. (**b**) MDC curvature plot of the α band along the momentum direction. (**c**) Dispersion of the α band extracted from **b** with the bare bands. (**d**) The gap values obtained from the symmetrized EDCs using the Dynes formula and EBC constant (λ) are plotted about cut1 ~ 5 in the inset. (**e**) Temperature dependent gap values and EBC constant. (**f**) Real part of the self-energy derived from the experimental band dispersions

To gain a deeper understanding of the phase transition in the α band of ML O-CoSe_2_, we examine its dispersion along with the 2D curvature analysis. Figure 4a depicts the α band dispersion along the $\stackrel{-}{\text{X}}$−$\stackrel{-}{{\Gamma }}$ direction with narrow energy and momentum ranges, revealing pronounced deviations around Fermi energy, known as kinks, which is not observed in the β band (Figure S10). To accentuate these deviations, we plotted the second derivative map of the α band along momentum direction (MDC curvature) in Fig. 4b [64]. These kinks signify modifications to the band dispersion induced by many-body interactions, serving as a key signature of strong EBC observed in other materials [9, 65–71]. The band trajectory of MDC in comparison with the bare band, is illustrated in Fig. 4c. The bare band is approximated using polynomial fitting aligning with the high binding energy (E_B_ < -80 meV) of the dispersion derived from the MDCs and passing through the Fermi wave vector(*k*_F_) (See Figure S11) [72, 73]. For the EBC analysis, we extract a single particle self-energy, Σ(**k**,ω), by comparing the bare band with the MDC curvatures, elucidating how collective modes and individual electronic excitations interact in the system. The self-energy is divided into real and imaginary parts, denoted as Σ′(**k**,ω) and Σ″(**k**,ω), representing modifications to the bare electronic dispersion and the electron lifetime, respectively. Building upon the aforementioned analysis, Fig. 4d, e depict the momentum- and temperature-dependent gap profiles, including EBC constant (Figure S12). The pseudo-gap and EBC constant exhibit a consistent trend depending on momentum and temperature, indicating a coupling between the phase transition and EBC in the α band. In Fig. 4f, we illustrate the real part of the self-energy using the equation $\sum\nolimits_\omega ^\prime { = \omega - \varepsilon _{{k_m}}^b}$, when $\omega$ is experimental binding energy and ${\epsilon }_{{k}_{m}}^{b}$ is binding energy of bare band at ${k}_{m}$($\omega$). A peak at 38.6 meV has been identified in the real part of the self-energies, indicating a characteristic energy of a boson mode. It is essential to measure the characteristic energy of magnetic resonances and phonon modes of this system or to adopt theoretical interpretations to offer a reasonable explanation of which boson (phonon or magnon) contributes to the many-body interaction in this system, leading to the kinks in the ARPES spectra. It would be intriguing to determine which boson is coupled in ML O-CoSe_2_ as a future work.

The reported TEM data in the literature shows that thick O-CoSe_2_ (bulk phase) lack collectively ordered states [42]. Therefore, the transition at the β band associated with FS nesting and 2 × 1 reconstruction is due to dimensional reduction. On the other hand, the other phase transition at the α band is coupled with EBC encompassing kink feature. It is noteworthy that a single material system rarely undergoes multiple phase transitions involving different kinds of many-body interactions. Therefore, our findings hold potential implications for exploring novel correlated electronic phases and their applications in related devices.

## Conclusions

In summary, the utilization of the state-of-the-art MBE growth technique, along with STM and ARPES measurements, has enabled the exploration of crystalline reconstruction and the corresponding renormalized electronic structure of ML O-CoSe_2_ for the first time. For the high temperature phase transition at 340 K, the STM results reveal an atomic modulation, including 2 × 1 superstructure, in the ground states of ML O-CoSe_2_, which is absent in its bulk counterpart. The pseudo-gap features in the nesting region of the CDW transition observed in the ARPES spectra exhibit a characteristic temperature dependence consistent with the STM results. The second phase transition is rather coupled with strong EBC which is revealed by a kink in the ARPES spectra. Therefore, ML O-CoSe_2_ exhibit two unique phase transitions simultaneously in their ground states with two different kinds of many-body correlations. Our findings suggest that O-CoSe_2_ is a suitable platform for investigating dimensional effects in non-vdW materials.

### Electronic supplementary material

Below is the link to the electronic supplementary material.

Supplementary Material 1

## Data Availability

The data that support the findings of this study are available from the corresponding author upon reasonable request.
