# Correction: Emergence of two distinct phase transitions in monolayer CoSe_2_ on graphene

**DOI:** 10.1186/s40580-024-00438-1

**Published:** 2024-08-31

**Authors:** Tae Gyu Rhee, Nguyen Huu Lam, Yeong Gwang Kim, Minseon Gu, Jinwoong Hwang, Aaron Bostwick, Sung-Kwan Mo, Seung-Hyun Chun, Jungdae Kim, Young Jun Chang, Byoung Ki Choi

**Affiliations:** 1 Department of Physics, University of Seoul, Seoul, 02504 Korea; 2 Department of Smart Cities, University of Seoul, Seoul, 02504 Korea; 3 Department of Physics, University of Ulsan, Ulsan, 44610 Korea; 4 Department of Physics, Institute of Quantum Convergence Technology, Kangwon National University, Chuncheon, 24341 Korea; 5 Advanced Light Source, Lawrence Berkeley National Laboratory, Berkeley, CA 94720 USA; 6 Department of Physics, Sejong University, Seoul, 05006 Korea; 7 Department of Intelligent Semiconductor Engineering, University of Seoul, Seoul, 02504 Korea

**Correction to: Nano Convergence (2024) 11:21** 10.1186/s40580-024-00427-4

Following publication of the original article [1], the author identified an error in Graphical Abstract and updated Acknowledgment section.

In Graphical abstract, there is a type on the unit ${{\varvec{E}}}_{{\varvec{B}}}({\varvec{e}}{\varvec{V}})$ which has been updated with this correction.

**Graphical abstract**


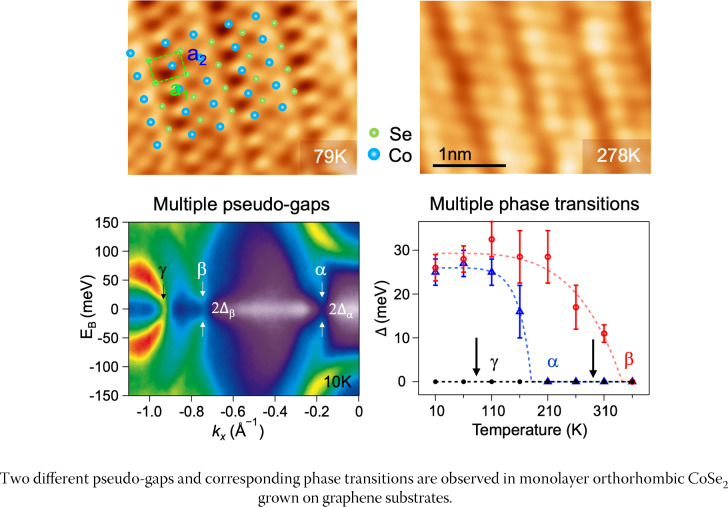


The work supported by MOLIT [Innovative Talent Education Program for Smart City] has been included in Acknowledgment statement.

**Acknowledgments**

This work is supported by National Research Foundation (NRF) grants funded by the Korean government (NRF-2019R1A6A1A11053838, NRF-2020R1A2C200373211, NRF-2021R1A6A3A14040322, RS-2023-00220471, RS-2023-00284081, RS-2023-00280346, RS-2023-00258359), [Innovative Talent Education Program for Smart City] by MOLIT, and Semiconductor R&D Support Project through the Gangwon Technopark (GWTP) funded by Gangwon Province (No. GWTP 2023-027). BKC was supported in part by an ALS Collaborative Postdoctoral Fellowship. This research used resources of the Advanced Light Source, which is a DOE Office of Science User Facility under contract no. DE-AC02-05CH11231. Experiments at PLS-II were supported in part by MSICT and POSTECH.

The original article has been corrected.
