# Supplementary material for: Emergence of two distinct phase transitions in monolayer CoSe2 on graphene
Source: Nano Converg. 2024 May 24;11:21. doi: 10.1186/s40580-024-00427-4 (PMC11126552; doi:10.1186/s40580-024-00427-4)
Supplement: Supplementary file 1 — Supplementary Material 1 [file 40580_2024_427_MOESM1_ESM.pdf]

Supplementary Information

Emergence of Two Distinct Phase Transitions in Monolayer $CoSe_2$ on Graphene

Tae Gyu Rhee[1,2,+], Nguyen Huu Lam[3,+], Yeong Gwang Kim[1,2], Minseon Gu[1], Jinwoong Hwang[4,5], Aaron Bostwick[5], Sung-Kwan Mo[5], Seung-Hyun Chun[6], Jungdae Kim[3,*], Young Jun Chang[1,2,7,*], Byoung Ki Choi[1,5,*]

[1] Department of Physics, University of Seoul, Seoul 02504, Korea.

[2] Department of Smart Cities, University of Seoul, Seoul 02504, Korea.

[3] Department of Physics, University of Ulsan, Ulsan 44610, Korea.

[4] Department of Physics and Institute of Quantum Convergence Technology, Kangwon National University, Chuncheon 24341, Korea.

[5] Advanced Light Source, Lawrence Berkeley National Laboratory, Berkeley, CA 94720, USA.

[6] Department of Physics, Sejong University, Seoul 05006, Korea.

[7] Department of Intelligent Semiconductor Engineering, University of Seoul, Seoul 02504, Korea.

+ Tae Gyu Rhee and Nguyen Huu Lam contributed equally to this work.
*Corresponding author: kimjd@ulsan.ac.kr, yjchang@uos.ac.kr, bkchoi@lbl.gov

KEYWORDS: transition metal chalcogenides, charge-density wave, electron-boson coupling, molecular beam epitaxy, angle-resolved photoemission spectroscopy, scanning tunneling microscopy

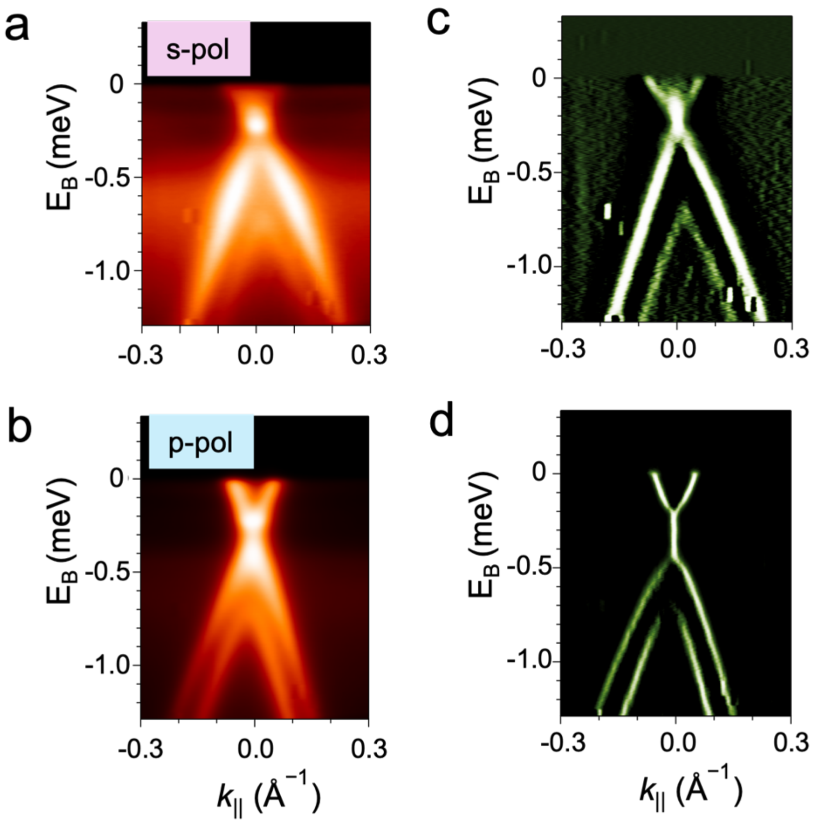

Supplementary Figure S1. ARPES maps of underlying BLG. (a,b) ARPES maps and (c,d) corresponding 2D curvature ARPES maps of BLG area taken from around K point of BLG for both polarizations, respectively.

The number of crossing bands directly correlates with the number of graphene layers on the graphene buffer layers, confirming the formation of BLG on the SiC substrates.[1,2]

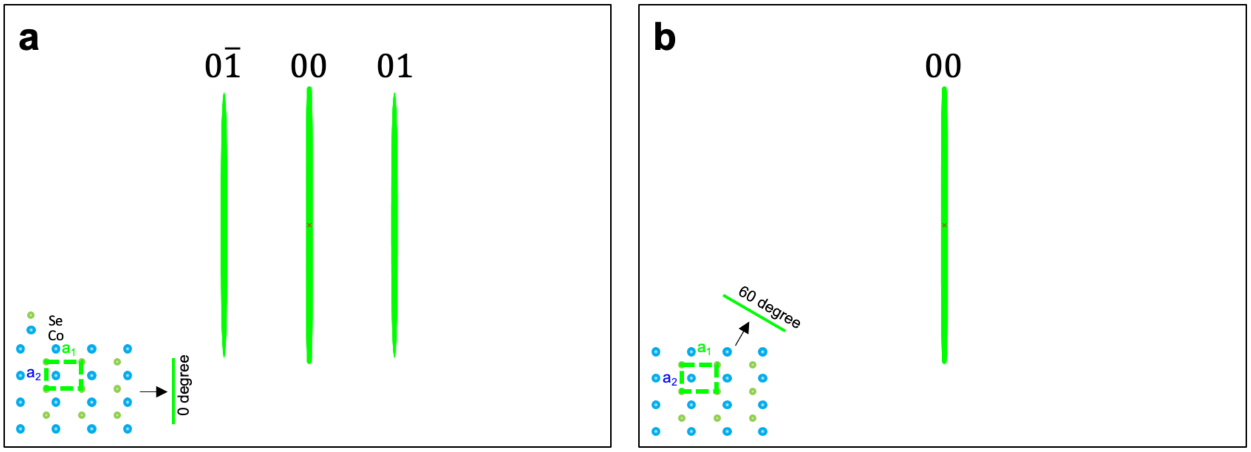

Supplementary Figure S2. Simulated electron diffraction image at different incident angles. (a,b) Simulated RHEED results with incident electrons at 0° and $\pm$120°, respectively.

Although ML O-$CoSe_2$ exhibits three distinct domains rotated by 120° with respect to each other, RHEED images display sharp peaks from only a single domain. To interpret these RHEED results, we conducted simulation of the electron diffraction of ML O-$CoSe_2$.[3] We introduced a 10 nm vacuum gap along the z-axis in unit cell structure and generated electron diffraction patterns in the in-plane directions. In Figure S2a, the simulated RHEED results exhibit line shapes that match the RHEED pattern of epitaxially grown O-$CoSe_2$ well. The distances between 00 line and 01 ($0\bar{1}$) lines are proportional to $\frac{2\pi}{a_2}$, originating from the crystalline periodicity. In Figure S2b, we simulated the electron diffraction with $\pm$120° rotated incident electron beams to validate the RHEED pattern of $\pm$120° rotated domains. In contrast to the simulated data with 0°, only 00 line exists. Therefore, our RHEED pattern in Figure 2b only shows the diffraction pattern of a single domain of ML O-$CoSe_2$ with 0°.

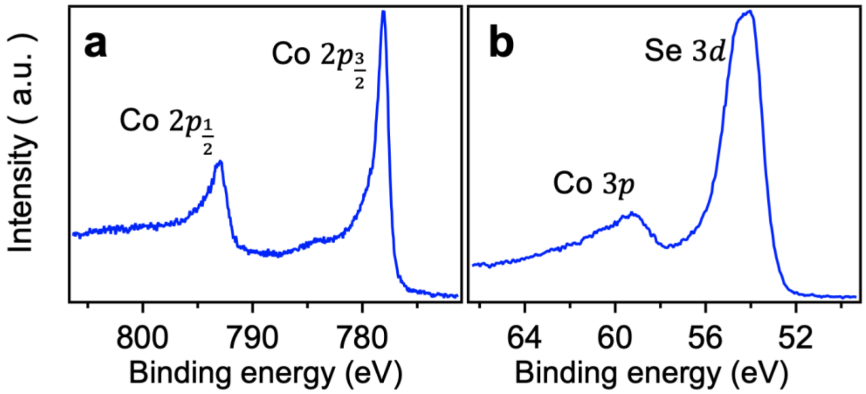

Supplementary Figure S3. XPS core level spectra of ML O-$CoSe_2$ Film. (a) XPS spectra of Co 2*p* orbital. (b) XPS spectra of Co 3*p* and Se 3*d* orbital.

We measured XPS spectra of the ML O-$CoSe_2$ film with photon energy of 1 keV to determine its atomic composition. In figure S3a, Co 2*p* doublet exhibits sharp and clear peaks, indicating ML O-$CoSe_2$ films are well-protected from oxidation or contamination through selenium capping method. The relative atomic ratio, extracted by (peak area)/(sensitivity factor), is $Co_1Se_{1.98}$, which is in good agreement with $CoSe_2$.

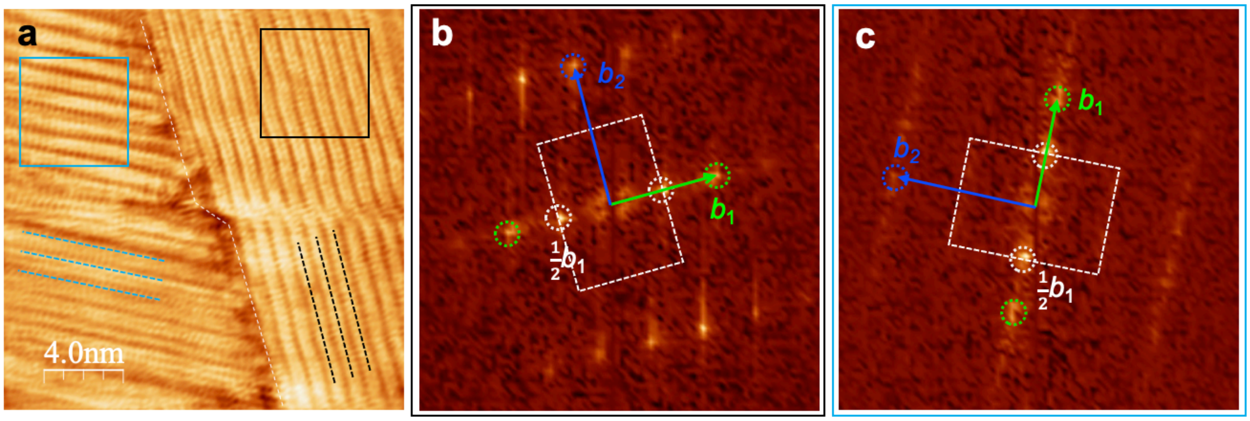

Supplementary Figure S4. 120 ° rotated domains. (a) Atomic image of two rotated domains sharing a boundary (white dashed line). (b-c) FFT images of the regions in the black and blue bashed squares on each rotated domain in **a**

We identified the region where two rotated domains coexist using STM. One domain is oriented approximately 120° rotated from the other. Both domains exhibit CDW ordering along their respective b_1 directions. However, near the domain boundary (white dashed line in figure S4a), atomic arrangement and CDW ordering become merged.

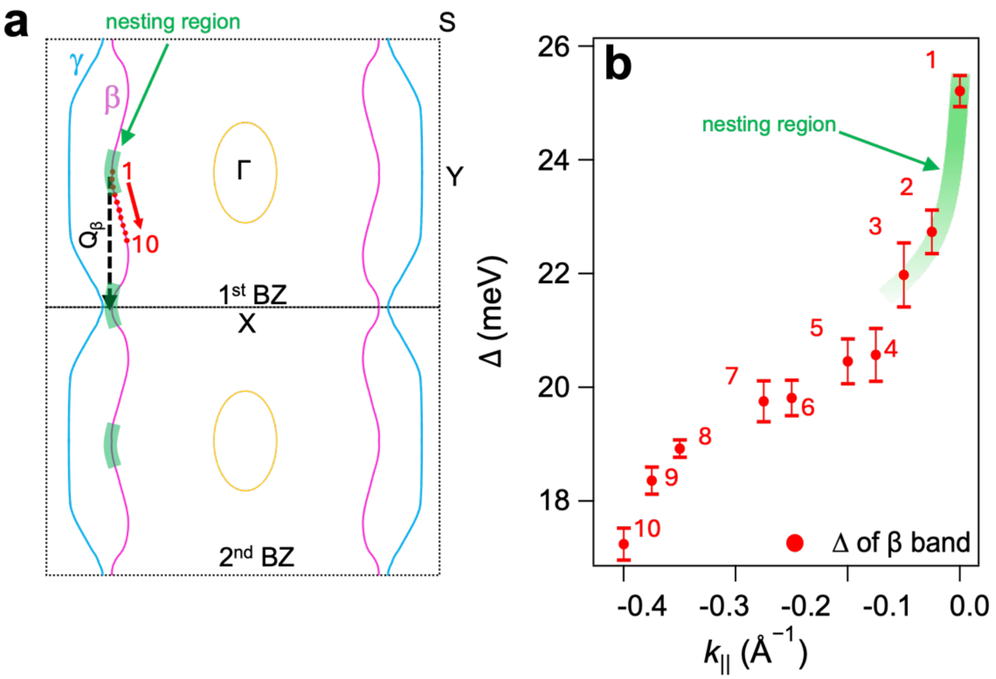

Supplementary Figure S5. Schematic of Fermi surface and gap profile of the β band. (a) A schematic of Fermi surface (FS) of ML O-$CoSe_2$. Q_β is nesting vector of β band. (b) Gap profiles of β band along the band trajectory, as indicated in a schematic of FS (point1~10). The nesting areas are marked by green shadow regions.

To pinpoint the nesting area (green shadow regions) and nesting vector (Q_β) of the β band, we illustrate a schematic of FS contour within 1st and 2nd BZ (Figure S5a). In the Figure S5b, the large gap region is marked by green shadow region, which is consistent with smallest spectral weight point in FS maps in Figure 2b.

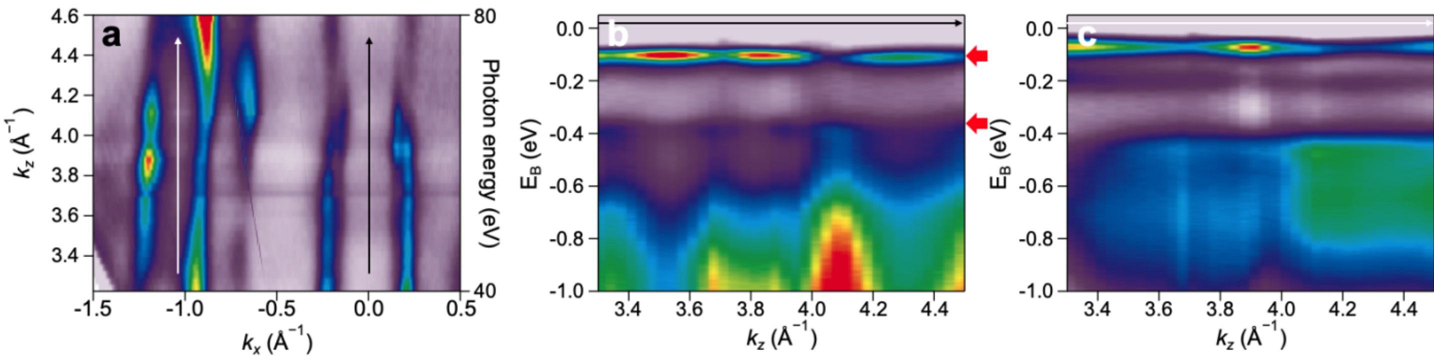

Supplementary Figure S6. ARPES photon energy scan. (a) Fermi surface contour on the k_{xz} plane. (b) Binding energy vs. k_z intensity map showing the absence of dispersion along k_z direction at the BZ center (black solid arrow in **a**) (c) and boundary (white solid arrow in **a**).

In a 2D system, the electronic structure exhibits two-dimensionality due to the absence of crystal periodicity along the z-axis. Consequently, ML O-$CoSe_2$ should manifest no dispersion along the k_z direction. Altering the photon energy during ARPES measurements allows the observation of the band dispersion along the k_z direction. This observation can be elucidated using the following equation, where E_k represents the kinetic energy of the emitted electron:

$$k_z = \frac{1}{\hbar}\sqrt{2m_e(E_k cos^2\theta + V_0)}$$

In Figure S6a, the FS contour on the k_{xz} plane is observed through scanning photon energy from 40 eV to 80 eV. All bands exhibit parallel lines along the k_z direction. In Figure S6b and S6c, all bands indicated with red arrow show no dispersion along the k_z direction, attributable to their two-dimensionality.

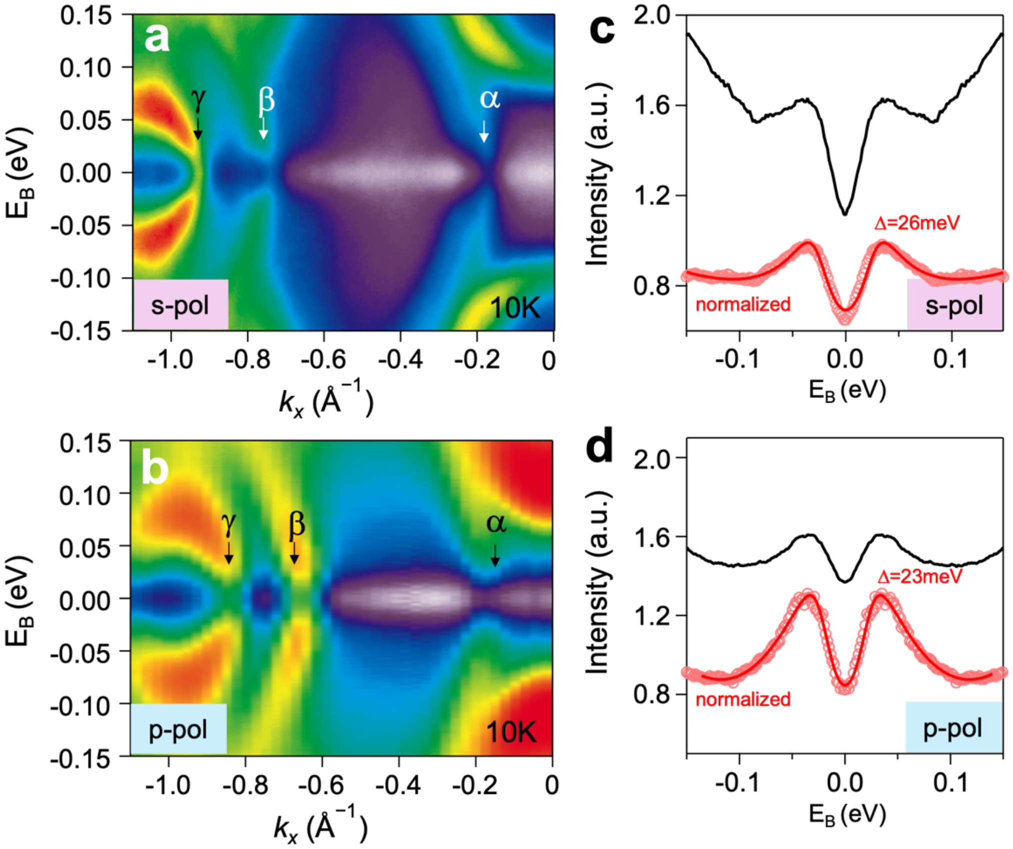

Supplementary Figure S7. Polarization dependence and kink analysis of β band in ML O-$CoSe_2$. (a,b) Symmetrized ARPES maps about the Fermi level, (c,d) EDC profiles of β band fitted by Dynes formula.

The ARPES-measured spectra can be significantly influenced by the matrix element (polarizations of the light sources) in principle, owing to the orbital structure.[4] In order to analyze the orbital character of β band, we plotted the ARPES maps for both s- and p-polarizations (Figure S7a,b). Although there are distinguishable differences in spectral weights, we observed similar band gaps by fitting the EDC profiles of β bands using Dynes formula (Figure S7c,d).

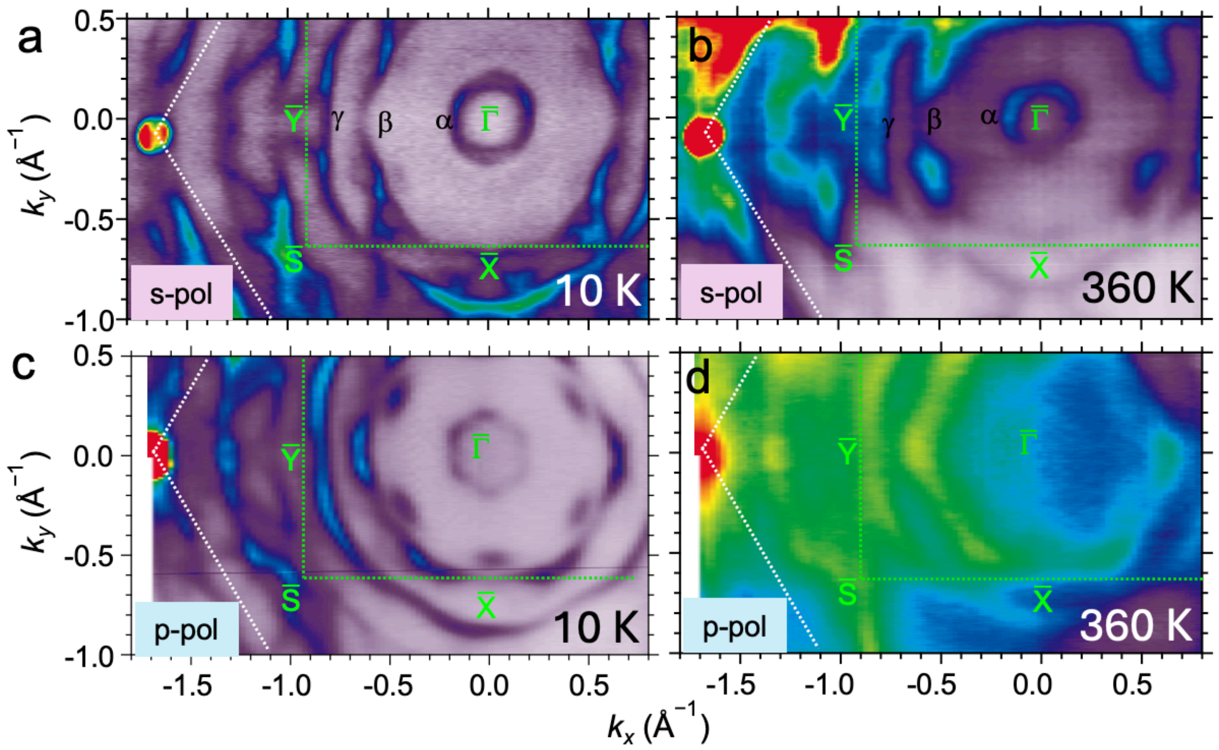

Supplementary Figure S8. Temperature dependent Fermi surface contour of ML O-$CoSe_2$. (a,b) Fermi surface contour with s-polarization and (c,d) p-polarization for 10 K and 360 K, respectively.

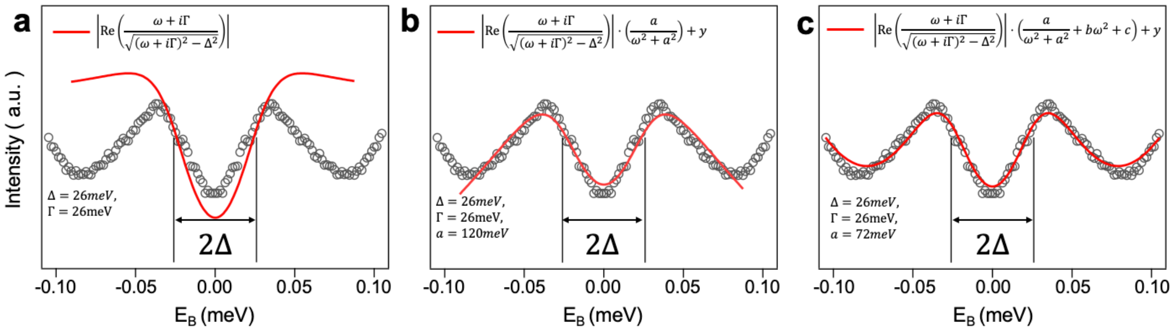

Supplementary Figure S9. Dynes formula. (a) Symmetrized spectra of alpha band at 60 K (black circles) and Dynes formula (red solid line) (b) Dynes formula with Lorentzian spectral function (c) Dynes formula with Lorentzian spectral and binomial background.

To characterize the pseudo gap resulting from CDW transition, we employ Dynes formula for gap fitting. Dynes formula, with Δ = 26 meV and Γ = 26 meV, effectively captures the pseudo gap near the Fermi level. However, since ARPES spectra generally adhere to a single-particle spectral function, Dynes formula alone falls short in describing the overall spectra.[5] This single-particle spectral function, in terms of binding energy, is represented by a Lorentzian curve. Subsequently, we refine the fitting of APRES results by multiplying it with a Lorentzian to accurately describe the spectra within the range of -80 meV < E_B < 80 meV. The $|E_B|$ > 80 meV, the spectra are predominantly influenced by binomial background. Ultimately, the pseudo gap can be extracted by fitting the spectra using Dynes formula multiplied with additional terms.

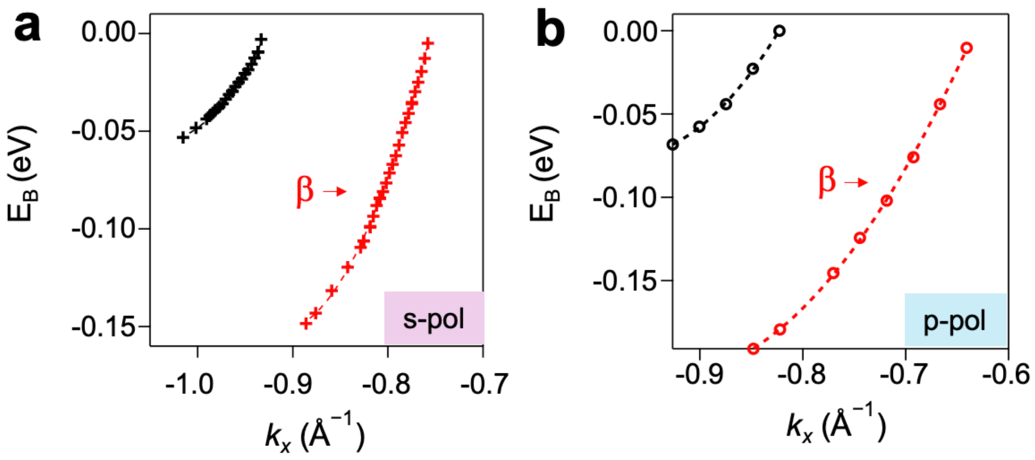

Supplementary Figure S10. Kink analysis of β band. (a,b) MDC profiles of β band with s- and p-polarizations, respectively.

Figure S10 displays MDC fitted lines of the β band for both polarizations. Notably, the β band does not exhibit kinks induced by electron-boson coupling.

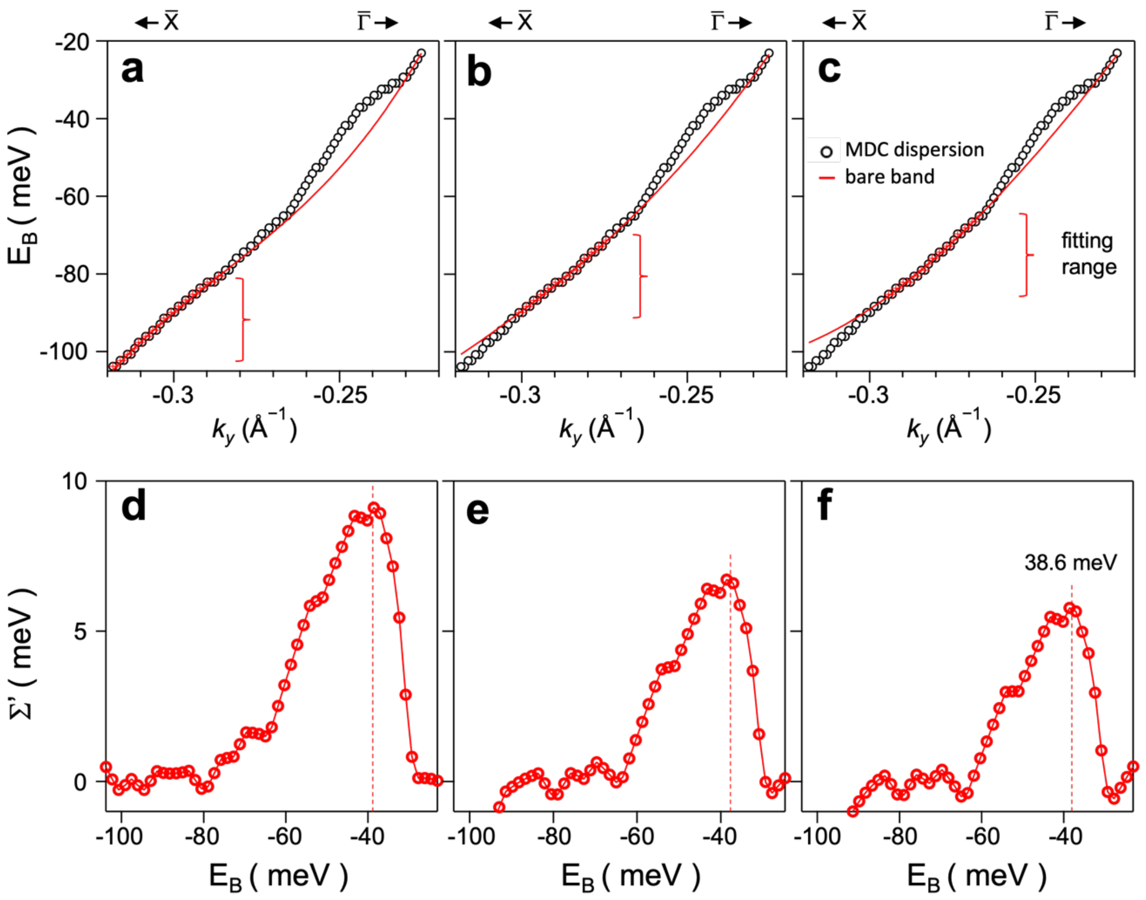

Supplementary Figure S11. Bare band fitting in EBC analysis. (a) Experimental MDC dispersion and estimated bare band by various cutoff energy range (meV) of -100 < E_B < -80, (b) -90 < E_B < -70, and (c) -80 < E_B < -60. (d-f) Real part of the renormalization energy of (a-c), respectively.

We plotted the bare band fitting and real part of the renormalized energy with various cutoff energy ranges to verify the reproducibility of the analysis. Regardless of cutoff energy ranges, the energy of peak in the real part of renormalized energy remains consistent.

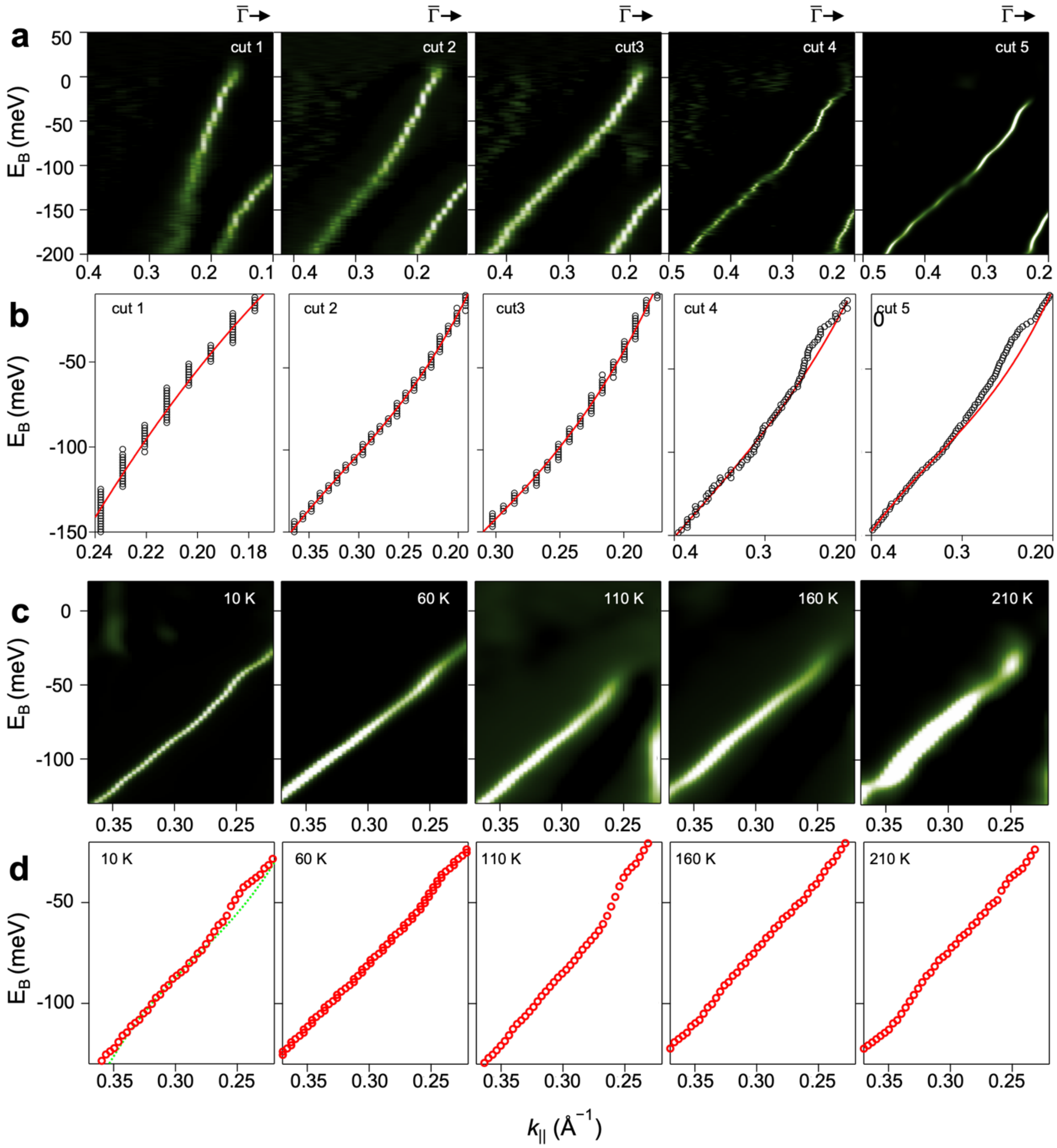

Supplementary Figure S12. Electron-boson coupling constant analysis of α bands depending on momentum and temperature. (a) MDC curvature plot of the α band taken along planar directions about cut1 ~ 5 in the Figure S5a and Figure 4d. (b) Dispersion of the α band extracted from **a** with the bare bands. (c) MDC curvature plot of the α band taken along $\bar{X}$–$\bar{\Gamma}$ direction about selected temperatures (d) Dispersion of the α band extracted from **c** with the bare bands.

REFERENCES

1. I. Razado-Colambo, J. Avila, D. Vignaud, S. Godey, X. Wallart, D. P. Woodruff, and M. C. Asensio, Sci. Rep. **8**, 10190 (2018).

2. M. Hajlaoui, H. Sediri, D. Pierucci, H. Henck, T. Phuphachong, M. G. Silly, L. A. De Vaulchier, F. Sirotti, Y. Guldner, R. Belkhou, and A. Ouerghi, Sci. Rep. **6**, 18791 (2016).

3. Y. Seto and M. Ohtsuka, J. Appl. Crystallogr. **55**, 397 (2022).

4. S. Moser, J. Electron Spectros. Relat. Phenomena **214**, 29 (2017).

5. I. Pletikosić, M. Kralj, M. Milun, and P. Pervan, Phys. Rev. B **85**, 155447 (2012).
